# Prevalence of emergency contraceptive pill use among Spanish adolescent girls and their family and psychological profiles

**DOI:** 10.1186/s12905-018-0560-x

**Published:** 2018-05-16

**Authors:** Antonia Jiménez-Iglesias, Carmen Moreno, Irene García-Moya, Francisco Rivera

**Affiliations:** 1 Departamento de Psicología Evolutiva y de la Educación, Universidad de Sevilla, C/ Camilo José Cela, s/n, 41018 Sevilla, Spain; 2 CRIPACC, School of Health and Social Work, University of Hertfordshire, Hatfield, UK; 3 Departamento de Psicología Experimental, Universidad de Sevilla, Sevilla, Spain

**Keywords:** Emergency contraceptive pill, Sexual intercourse, Adolescence, Girls, Family, Well-being

## Abstract

**Background:**

Adolescent girls’ family context and psychological characteristics play important roles in their sexual behavior, including the use of the emergency contraceptive pill (ECP). This study aims to (1) determine the prevalence of ECP use among girls who have had sexual intercourse and (2) comparatively analyze their family and psychological profiles according to whether they have used ECPs.

**Methods:**

The sample of 1735 Spanish girls aged 15 to 18 came from a representative sample of the 2014 edition of the Health Behaviour in School-aged Children (HBSC) study. Of this sample, 398 girls had sexual intercourse and reported their ECP use. Data collection for the HBSC study was performed through an online questionnaire to which adolescents responded anonymously in school. Data analyses were descriptive and bivariate and were performed with the statistical program IBM SPSS Statistics 23.

**Results:**

The results demonstrated that 30.65% of girls who had sexual intercourse used ECPs. Noticeable differences in paternal knowledge and communication with the father were observed between girls who used the ECP at least once and those who did not use it. In contrast, differences between girls who used the ECP once and those who used it twice or more were pronounced with regard to parental knowledge, communication with parents, maternal affection, life satisfaction, sense of coherence and depression.

**Conclusions:**

This work demonstrates a high prevalence of ECP use and a more positive family and psychological profile for girls who used ECP once compared with those who used it twice or more.

## Background

Emergency contraception (EC) refers to methods of contraception that any woman of reproductive age may need in cases of unprotected sexual intercourse, contraceptive failure, incorrect contraceptive use, or sexual assault. EC is recommended within five days after sexual intercourse (although it is more effective when used as soon as possible), when EC can prevent an unwanted pregnancy but cannot cause an abortion or damage a developing embryo [1]. Therefore, EC is an opportunity that must not replace usual contraceptive methods and that does not protect against sexually transmitted diseases [2].

Among existing EC methods, this work focuses on the study of the emergency contraceptive pill (ECP). In Spain, the distribution of the levonorgestrel ECP in pharmacies without a prescription has been authorized since 2009, although it was marketed for the first time in 2001. The ulipristal acetate ECP was first marketed in 2009 [2], and its distribution without prescription in pharmacies has been authorized since 2015. In certain Spanish regions, ECP use has no cost for users in public health centers, or its cost is partially or completely refunded if it is purchased with a prescription in pharmacies [3]. This makes it more accessible, especially for the most vulnerable populations, such as adolescent girls, women of low socio-economic status and immigrants [4]. Furthermore, because the ECP prevents unwanted pregnancies, it is especially important in adolescence because an unwanted pregnancy at this stage of life has more negative effects than in adulthood. For the above reasons, many experts assert that access to ECPs must be straightforward for younger girls but that it is necessary to properly inform them beforehand regarding their use [2, 5].

Easy access to ECPs does not entail decreased use of other contraceptive methods. International data from 42 countries in Europe and North America from the 2014 edition in the Health Behaviour in School-aged Children (HBSC) study show that the percentage of Spanish adolescents who used condoms during their last sexual intercourse is high, higher than the use of contraceptive pills. It is especially higher for girls, Spain being the *only* country in the study in which the percentage of condom use was higher for girls than for boys [6]. One study, which analyzed data on ECP use at last sexual intercourse from eleven countries of the 2006 edition of the HBSC study (Flemish Belgium, Bulgaria, England, Finland, France, Greece, Hungary, Latvia, Sweden, Ukraine, Wales), found that France had the highest percentage of ECP use, probably due to easier access to ECPs. However, this finding did not entail that girls did not use other methods to protect themselves against pregnancy given that the percentages of condom use and/or contraceptive pill use were also high [7]. In short, it seems that adolescents with better access to EC use it when they need it, but they do not modify their usual method of contraception and/or they do not increase their sexual risk behavior [8].

Several researchers have analyzed the profiles of ECP users, who are usually adolescent girls and young women [9–12] whose main motive in requesting ECPs is an accident with the male condom, although non-use of contraceptive methods is also indicated by a small percentage of respondents [9, 10, 12, 13].

Despite common EC use, knowledge of EC seems low in adolescence [14]. Therefore, the information that adolescents receive about ECPs and sexuality in general must be improved. Regarding parents as a source of information, one study found that for a third of participants, mothers were the first source of reproductive information. Although fathers were not a first source of reproductive information, some participants indicated that fathers were available to guide them with regard to romantic relationships [15]. However, despite good relationships with their mother and father, most girls do not sufficiently trust their parents to request information from them or to talk to them about their sexual experiences because girls see these as intimate issues. Girls are also reluctant to ask their parents for help in requesting ECPs because they think that this may not be accepted or understood [13]. Nevertheless, girls often want to talk with their parents about sexuality and romantic relationships [15].

The aforementioned studies emphasize the relevance of family context to healthy sexual development. Among the relevant family dimensions, parental warmth and emotional closeness in the parent-child relationship seem to be related to healthier sexual behaviors, especially for girls [16]. These close parent-child relationships make communication about romantic and sexual relationships more likely, although a close relationship with parents does not guarantee that adolescents will talk about these issues, and discussing them can be effective regardless of whether or not the relationship is close [15]. Furthermore, parent-child communication about issues related to sex is more likely to encourage healthy sexual development and to decrease sexual risk behaviors when parents are open and skilled and they feel comfortable in these discussions [17]. Accordingly, if parents communicate with their adolescent children concerning contraceptive methods and preventing sexually transmitted diseases, adolescents will be more likely to use contraceptive methods [18]. Parental knowledge, a dimension related to communication, is also associated positively with contraceptive use and is very important for healthy sexual development [19]. In fact, adolescents, especially girls, whose parents are more knowledgeable about their friends and activities report lower levels of sexual risk behaviors [20].

Finally, the relationship between sexual behavior and well-being is important since sexual behavior can be both a risk factor and an asset for adolescent well-being. Thus, adolescents who have experience in sexual intercourse and for whom it is normative in their group report better well-being [21], especially boys [22], whereas adolescents who are less sexually active than their peer group report less well-being [21]. Moreover, depression can lead to risky behaviors in a sexual situation, such as lower condom use, especially in girls and women [23]. A strong sense of coherence (SOC), a predictor of mental health, is associated with less involvement in health risk behaviors [24].

Therefore, adolescent girls’ family context and their psychological characteristics are important factors for their sexual behavior. However, the role of these factors in ECP use and the prevalence of ECP use among adolescent girls have rarely been studied. The above findings, together with the relevance of this topic, highlight the need for this study.

The aims of this study were (1) to determine the prevalence of ECP use among adolescent girls who have had sexual intercourse; (2) to comparatively analyze the family and psychological profile of girls who have used ECP at least once compared with those who have had sexual intercourse and have never used it and of girls who have used ECP once compared with those who have used it twice or more.

## Methods

### Study design

The present work takes its data from the 2014 edition of the HBSC study in Spain, which is coordinated by the Spanish HBSC team. The international HBSC study, a World Health Organization collaborative cross-national survey, collects data about health behaviors, social contexts, and the health and well-being of adolescents every four years in more than 40 countries and regions across Europe and North America including Spain. The last edition of the study was in 2013/2014, and the first survey was in 1983/1984 in five countries [6]. This study uses a cross-sectional design.

### Participants

Participants came from the HBSC study’s representative sample of the Spanish school population aged 11 to 18 years, selected by a random multistage sampling stratified by conglomerates considering adolescents’ age, habitat (rural or urban) and type of school (public or private) [25].

The present study focused on girls aged 15–16 (63.46%) and 17–18 years (36.54%), a total of 1735 girls who could answer all variables analyzed in this study. Of these, the final sample in this study consisted of 398 girls who had responded to the ECP question. Data for the remaining girls were missing for the following reasons: 1073 girls did not respond due to not having had sexual intercourse, and 34 girls did not respond due to not knowing/not responding. Additionally, the data of 230 girls were considered missing from the system.

### Measures

The instrument used in this work was the questionnaire of the HBSC study [26]. Specifically, variables analyzed are described in Table 1 and are listed below.

- Sexual behavior variable: ECP*.*
- Family dimensions: maternal and paternal knowledge [27], communication with the mother and the father, and maternal and paternal affection [28].
- Psychological variables: life satisfaction [29], sense of coherence [30], and depression [31].

**Table 1:** Variables from the questionnaire of the HBSC study analyzed in this study

Sexual behavior variable	Brief description	Response options
ECP	An item developed by the Spanish HBSC team that assesses the number of times that girls have used ECPs in their lives.	*No, never*, *Yes, once*, *Yes, 2 times*, and *Yes, 3 times or more.*
Family dimensions	Brief description	Response rank
Maternal and paternal knowledge	Scales taken from the parental knowledge scale of Brown, Mounts, Lamborn, and Steinberg [27], which asks adolescents what their father/mother knows about their life outside of home.	From 0 (*low knowledge*) to 2 (*high knowledge*).
Communication with the mother and the father	Two items developed by the international HBSC study from its beginning. They assess adolescents’ perception about ease of communication with their mother/father.	From 0 (*very difficult*) to 3 (*very easy*).
Maternal and paternal affection	Scales taken from one of the Parental Bonding Instrument-Brief Current form (PBI-BC) subscales of Klimidis, Minas, and Ata [28] that ask adolescents about how their father/mother demonstrates affection to them.	From 0 (*low affection*) to 2 (*high affection*).
Psychological variables	Brief description	Response rank
Life satisfaction	Taken from the Satisfaction With Life Scale [29], a scale composed of 5 items to assess global life satisfaction, one of the components of subjective well-being.	From 1 to 7 (higher values indicate higher life satisfaction).
Sense of coherence	A version of 13 items (SOC-13) of the Orientation with Life Questionnaire [30], which assesses different aspects related to salutogenesis, an approach aimed at identifying and promoting the so-called salutogenic factors, i.e., those factors that foster health and well-being.	From 1 to 7 (higher values indicate a greater sense of coherence).
Depression	Taken from the Center for Epidemiologic Studies Depression Scale*,* CES-D12 [31], composed of 12 items that assess depressive symptoms in adolescence.	From 1 to 4 (higher values indicate higher level of depression).

### Data collection

Data collection for the HBSC study, which occurred throughout the entirety of Spain from March to December 2014, was performed online through a questionnaire that was accessed from computers connected to the Internet in schools. If the schools had difficulties with Internet access or with computers, tablets were used. This data collection fulfilled the requirements of the international network of the HBSC study [25], which establish that adolescents must respond to the questionnaire, they must do so in a school context, and the anonymity of their responses must be assured [6].

### Data analysis

Data analyses, performed with the statistical program IBM SPSS 23 (IBM Corp., Armonk, NY) in this work, differed depending on the aims of the study. For aim (1), the analyses were descriptive to determine the prevalence of ECP use. In the case of aim (2), the analyses were descriptive and bivariate to examine differences related to the family and psychological profile. Specifically, this included two sets of analyses: a comparison between girls who had not used ECPs (value *No, never*) and girls who had used ECPs at least once (sum of values *Yes, once*, *Yes, two times* and *Yes, three times or more*) and a comparison between girls who had used the ECP once (value *Yes, once*) and girls who had used ECPs twice or more (sum of values *Yes, two times* and *Yes, three times or more*). For this second aim, the significance test was Student’s *t* for independent samples, considered significant if *p* < .05, and the effect size test was Cohen’s *d*, in which, based on his criteria, *d* values were considered negligible (less than 0.20), small (from 0.20 to 0.499), medium (from 0.50 to 0.799) or large (0.80 or greater) [32].

## Results

The prevalence of ECP use was high. 122 girls had used the ECP sometime in their lives (19.85% once and 10.80% twice or more; total prevalence of 30.65%) compared with 276 girls (69.35%) who had had sexual intercourse and had never used it.

Regarding family dimensions, as observed in Table 2, differences between girls who had not used ECPs and girls who had used ECPs at least once were significant and had a small effect size for paternal knowledge. The differences between these two groups were not significant but had a small effect size for communication with the father. Both family dimensions (paternal knowledge and communication with the father) had higher mean values among girls who had never used ECPs.

**Table 2:** Family dimensions and ECP

Family dimensions	ECP						Significance test and effect size test
	No, never			Yes, at least once			
	*N*	*M*	*SD*	*N*	*M*	*SD*	
Maternal knowledge	248	1.71	0.39	104	1.67	0.43	*t*(350) = 0.911. *p* = .363 *d* = 0.10
Paternal knowledge	194	1.41	0.54	80	1.22	0.59	*t*(272) = 2.545, *p* = .011 *d* = 0.34
Communication with the mother	247	1.80	0.94	104	1.89	0.91	*t*(349) = −0.853, *p* = .394 *d* = 0.10
Communication with the father	197	1.28	0.91	80	1.10	0.92	*t*(275) = 1.482, *p* = .140 *d* = 0.20
Maternal affection	246	1.58	0.48	102	1.52	0.49	*t*(346) = 0.979, *p* = .328 *d* = 0.12
Paternal affection	196	1.35	0.59	76	1.27	0.55	*t*(270) = 1.004, *p* = .316 *d* = 0.14
Family dimensions	ECP						Significance test and effect size test
	Yes, once			Yes, two times or more			
	*N*	*M*	*SD*	*N*	*M*	*SD*	
Maternal knowledge	68	1.73	0.36	36	1.55	0.53	*t*(52.290) = 1.865, *p* = .068 *d* = 0.43
Paternal knowledge	49	1.30	0.51	31	1.09	0.69	*t*(50.751) = 1.474, *p* = .147 *d* = 0.36
Communication with the mother	68	1.97	0.86	36	1.75	1.00	*t*(102) = 1.174, *p* = .243 *d* = 0.24
Communication with the father	49	1.20	0.91	31	0.94	0.93	*t*(78) = 1.274, *p* = .206 *d* = 0.28
Maternal affection	66	1.63	0.42	36	1.33	0.57	*t*(56.070) = 2.850, *p* = .006 *d* = 0.63
Paternal affection	48	1.29	0.54	28	1.24	0.58	*t*(74) = 0.344, *p* = .732 *d* = 0.09

In contrast, differences between girls who had used ECP once and girls who had used ECPs twice or more were significant and had a medium effect size for maternal affection. These differences were not significant but had small effect sizes for maternal and paternal knowledge and for communication with the mother and with the father. Mean values of these family dimensions (maternal affection, maternal and paternal knowledge and communication with the mother and with the father) were higher among girls who had used ECP once compared with girls who had used ECPs twice or more (see Table 2).

With respect to psychological variables (see Table 3), only the differences between girls who had used the ECP once and girls who had used ECPs twice or more were significant and had a small effect size for SOC. However, the differences were not significant but had small effect sizes for life satisfaction and depression. The mean values of life satisfaction and SOC were higher in girls who had used ECP once, whereas the mean value of depression was higher in girls who had used ECPs twice or more.

**Table 3:** Psychological variables and ECP

Psychological variables	ECP						Significance test and effect size test
	No, never			Yes, at least once			
	*N*	*M*	*SD*	*N*	*M*	*SD*	
Life satisfaction	260	4.66	1.44	118	4.60	1.45	*t*(376) = 0.388, *p* = .698 *d* = 0.04
SOC	250	4.07	0.88	110	3.91	0.90	*t*(358) = 1.565, *p* = .119 *d* = 0.18
Depression	248	2.03	0.54	104	2.08	0.55	*t*(350) = −0.764, *p* = .445 *d* = 0.09
Psychological variables	ECP						Significance test and effect size test
	Yes, once			Yes, two times or more			
	*N*	*M*	*SD*	*N*	*M*	*SD*	
Life satisfaction	76	4.71	1.39	42	4.41	1.56	*t*(116) = 1.060, *p* = .292 *d* = 0.21
SOC	69	4.06	0.81	41	3.65	0.99	*t*(108) = 2.350, *p* = .021 *d* = 0.47
Depression	65	2.00	0.41	39	2.20	0.72	*t*(53.220) = −1.598, *p* = .116 *d* = 0.38

## Discussion

This work had two aims: (1) to examine the prevalence of ECP use in adolescent girls who have had sexual intercourse and (2) to analyze the family and psychological profile of girls who have used the ECP at least once compared with girls who have never used it as well as of girls who have used the ECP once compared with girls who have used it twice or more. Regarding this second aim, it is necessary to indicate that the results discussed are those results that obtained noticeable effect size values regardless of whether or not differences were significant considering their *p* values since in some variables, differences were not significant. This is probably due to the lack of sample size in some crosses, but the effect size was noticeable.

The prevalence of ECP use among Spanish girls aged 15 to 18 years was high given that almost one in three adolescents (30.65%) who had sexual intercourse used ECPs. This finding agrees with another study in Spain in which 28.2% of adolescent girls aged 14 to 18 who had sexual intercourse used ECPs [33]. Nevertheless, condom use is also high in Spain [6]. This prevalence of use among adolescent girls who have had sexual intercourse varies in different studies from other countries; for example, 8.2% of Greek girls with a mean age of 14.7 [34] and 20% of Swiss girls aged 16 to 20 [35] have used ECPs.

Regarding adolescent girls’ family profiles, differences found between girls who had not used ECPs and girls who had used ECP at least once occurred only in paternal knowledge and communication with the father. Girls who had not used ECPs, compared with those who had used ECP at least once, reported that their fathers were more knowledgeable about their lives away from home and that communication with them was easier, which is likely to increase paternal knowledge. These results seem concordant with the results found in a previous study, in which higher paternal knowledge of their children’s friends and activities indicated lower levels of sexual risk behaviors, particularly in girls. Furthermore, fathers may react to their adolescent children’s risk behaviors with greater supervision and consequent knowledge of their lives [20], which could have protected girls in the present study from encountering risk situations that may lead to ECP use.

With respect to girls who used the ECP once and girls who used it twice or more, differences between them in family dimensions were greater given that parental knowledge, communication with parents and maternal affection were higher among girls who used the ECP once. This finding indicates that vulnerability is reflected not in the use of ECP but in using it more than one time. As other researchers have observed, family plays an important role in healthy sexual development, especially through the dimensions analyzed in this work, such as emotional closeness [16], parent-child communication about issues related to sex [17, 18] or parental knowledge [19]. In this way, parents have the opportunity and the necessary skill to influence their adolescent children’s decision-making about their sexual behaviors [18], in this case regarding responsible contraceptive use and the option to request ECP if contraceptives fail. The above findings suggest that professionals should encourage parents to discuss sexuality and romantic relationships with their children, avoiding judgmental attitudes, to improve their adolescent children’s decisions and behaviors [15] and that it is important that these discussions begin in childhood. If parents have not spoken openly about sexuality throughout the child’s life, doing so in adolescence may be too late [13].

In relation to adolescent girls’ psychological profiles, no differences were observed between girls who used the ECP at least once and girls who never used it. Differences were only found between girls who used the ECP once and girls who used it twice or more. Life satisfaction and SOC were higher in girls who used the ECP once, whereas depression was higher in girls who used ECPs twice or more. The relation between high life satisfaction and one-time ECP use shows that these girls have good psychological resources that allow them to make effective decisions in important moments; in this sense, ECP availability serves as an asset to adolescents’ well-being [21]. However, the relationship between increased depressive symptoms and ECP use twice or more shows a different result: on the one hand, the recurring behavior of ECP use may entail a risk for girls’ well-being [21]; on the other hand, it is also possible that depression reduces the initiative necessary for self-care behaviors, such as condom use [23]. Additionally, greater SOC associated with one-time ECP use exposes an expected relationship considering that high SOC is related to a greater capacity to understand and to give meaning to daily activities and the presence of resources to face stressful situations in life [36], which usually leads to lower involvement in health risk behavior [24].

Finally, the fact that this study does not allow for the exploration of the reasons for girls’ ECP use or non-use is a limitation to consider in interpreting the results of this work. Another limitation of this work is its cross-sectional design, which does not allow for the analysis of causal relationships between the analyzed variables. A final limitation is related to the sample of this study, which was a small group of the representative sample of girls in the HBSC study. However, this was mainly caused by the characteristics of the sample (the majority of the girls did not respond to the ECP question because they had not had sexual intercourse). Furthermore, 34 girls did not respond to the ECP question despite it being presented to them, probably because this is a sensitive question. On a related matter, the small size of the sample affected the analyses used in this study since it did not allow the performance of multivariate analyses. Nevertheless, this work has also strengths since it is based on a representative sample of Spanish adolescent schoolgirls aged 15 to 18 years, and it broadens the knowledge about an area of ECP use that has been little researched.

## Conclusions

This work reports that the prevalence of ECP use is high among Spanish girls aged 15 to 18 years who have had sexual intercourse and presents a more precise and positive image of these girls. This study demonstrates a better family and psychological profile for girls who have used the ECP once compared to girls who have used ECPs twice or more. The above emphasizes the importance of promoting an adequate affective-sexual education for adolescent boys and girls in their family and school contexts [2, 37]. Likewise, the finding that adolescents who used the ECP twice or more showed the most negative profiles may hint at the importance of providing adolescents with sexual and reproductive health services, including contraceptive information and services that they can access without requiring parental or guardian authorization, as recommended by the World Health Organization [37]. Therefore, the results of the present work may have important implications for public health and intervention in the adolescent girl population.

## Abbreviations

EC: Emergency contraception
ECP: Emergency contraceptive pill
HBSC: Health Behaviour in School-aged Children
SOC: Sense of coherence
